# Combined Effects of Simulated Gastric Acid, Coffee Immersion, and Cleaning Procedures on the Surface Topography and Optical Properties of Flowable Resin Composites

**DOI:** 10.3390/polym18151827

**Published:** 2026-07-26

**Authors:** Lena Bal, Cangül Keskin, Gökçe Naz Cömert, Osman Fatih Aydın, Fatma Öztürk Tunçel

**Affiliations:** 1 Department of Restorative Dentistry, Faculty of Dentistry, Ankara Medipol University, 06570 Ankara, Turkey; osman.aydin@ankaramedipol.edu.tr; 2 Department of Endodontics, Faculty of Dentistry, 19 Mayıs University, 55270 Ankara, Turkey; canglkarabulut@gmail.com; 3 Department of Prosthodontic Dentistry, Faculty of Dentistry, Ankara Medipol University, 06570 Ankara, Turkey; gokce.comert@ankaramedipol.edu.tr; 4 Department of Restorative Dentistry, Faculty of Dentistry, Health Sciences University, 06018 Ankara, Turkey; fatma.ozturk1@sbu.edu.tr

**Keywords:** flowable resin composite, gastric acid, dental erosion, surface roughness, color stability

## Abstract

The durability of dental restorations against erosive and staining challenges is crucial for long-term clinical success. Therefore, this in vitro study aimed to investigate the combined effects of sequential simulated gastric acid exposure, coffee immersion, and different cleaning procedures on the surface roughness and color stability of four contemporary flowable resin composites with different filler and matrix characteristics. Ninety-six disk-shaped specimens were fabricated from four resin composites: Omnichroma Flow, G-ænial Universal Injectable, Universal Flo, and EverX Flow. Baseline surface roughness and color coordinates were recorded. Specimens were immersed in 0.06 M hydrochloric acid for 48 h to simulate gastric acid exposure and subsequently immersed in coffee solution for 24 h to simulate staining. Color measurements were performed at baseline, after sequential gastric acid–coffee exposure, and after cleaning procedures. ΔE_0_ was calculated between baseline and post-exposure values, whereas ΔE_1_ was calculated between post-exposure and post-cleaning values. Specimens were then allocated to three cleaning subgroups: toothbrushing (BR), water flosser (WF) and mouthrinse (MR). Final surface roughness and color changes were evaluated. Data were analyzed using mixed and factorial ANOVA tests. Sequential gastric acid–coffee exposure significantly increased surface roughness in all materials. Omnichroma Flow showed the lowest roughness values, whereas G-ænial Universal Injectable demonstrated the highest post-treatment roughness, particularly after toothbrushing. Toothbrushing generally caused greater roughness increases than other cleaning procedures. Material type significantly affected color stability, with Universal Flo showing the greatest discoloration and Omnichroma Flow the lowest color change. Surface roughness and color stability were significantly influenced by sequential gastric acid–coffee exposure and cleaning procedures in a material-dependent manner.

## 1. Introduction

Resin-based composites are widely used in modern restorative dentistry owing to their superior esthetics, reliable adhesive bonding, and support for minimally invasive techniques. Particularly, flowable resin composites have seen broadened clinical utility due to their enhanced handling, excellent cavity wall adaptation, and extended indications for both anterior and posterior restorations [1,2]. Most of these materials can be readily used in patients with systemic diseases.

Gastroesophageal reflux disease (GERD) is a chronic condition characterized by the retrograde flow of gastric contents into the esophagus and, occasionally, the oral cavity. Repeated exposure to gastric acid may reduce oral pH to erosive levels, leading to progressive degradation of enamel, dentin, and even restorative materials [3]. As GERD often progresses silently or with non-specific symptoms, its oral manifestations, such as increased surface roughness, discoloration, and restoration failure, may serve as the earliest clinical indicators of the disease, highlighting the importance of dental professionals in early detection and management [4,5].

Dental restorative materials are not only exposed to gastric acid in the oral environment but also to various acidic foods and drinks consumed daily. Although saliva plays an important role in buffering and maintaining oral pH balance, restorative composites must exhibit superior resistance to withstand such erosive and abrasive challenges without significant degradation of their surfaces and mechanical properties [3,6,7,8]. When the buffering capacity of saliva decreases and composite restorations are exposed to an acidic pH for extended periods, hydrolysis of the methacrylate ester bonds within the resin matrix occurs. This degradation process adversely affects the surface roughness and microhardness of the restorations, ultimately compromising their long-term clinical performance [9,10,11].

Recent developments in flowable resin composites, including injectable, highly filled, single-shade, and fiber-reinforced formulations, have improved their handling characteristics, mechanical performance, and clinical applicability [2,12,13]. Owing to their low viscosity, enhanced adaptation, and esthetic potential, flowable composites are increasingly used in minimally invasive restorative procedures, cervical and buccal defects, small anterior and posterior restorations, and areas requiring precise adaptation [14,15].

These indications are particularly relevant in patients exposed to erosive challenges, such as gastroesophageal reflux disease, where early surface defects and restoration margins may be repeatedly subjected to acidic and staining conditions.

Previous studies have separately evaluated the effects of acidic media on the surface characteristics of resin-based and flowable composites [3,16]. In addition, staining solutions, coffee immersion, and toothbrushing abrasion have been investigated as individual challenges affecting the color stability, surface roughness, and gloss of resin-based composites [17,18,19]. However, limited information is available regarding the sequential and combined effects of intrinsic acid exposure, coffee staining, and subsequent cleaning procedures on contemporary flowable resin composites. This gap is clinically relevant because restorations in patients with GERD may be exposed not only to recurrent gastric acid but also to dietary staining agents and daily oral hygiene procedures [3,5]. Therefore, evaluating these challenges in a sequential protocol may better reflect the combined clinical conditions encountered by restorations in patients with erosive risk.

Moreover, modern flowable composites differ considerably in filler content, filler morphology, resin matrix composition, and reinforcement strategies, including supra-nano-filled single-shade systems, Bis-GMA-free nanohybrid formulations, highly filled injectable composites with full-coverage silane coating, and short fiber-reinforced flowable composites [2,12,13]. These structural differences may influence their resistance to surface degradation and discoloration under combined erosive–staining and cleaning challenges.

Therefore, the uniqueness of the present study lies in evaluating four contemporary flowable resin composites with markedly different formulations under a sequential simulated gastric acid–coffee exposure protocol followed by clinically relevant cleaning procedures, including toothbrushing, mouthrinse immersion, and water flosser application [2]. The null hypothesis tested was that the surface roughness and color stability of the flowable resin composites would be affected neither by the chemical composition of the material nor by the type of clinical cleaning procedure following simulated gastric acid and coffee exposure.

## 2. Materials and Methods

### 2.1. Sample Size and Preparation

Four different flowable resin composites indicated for direct restorative procedures, Omnichroma Flow (OMN), Injectable Flow (INJ), Universal Flo (UNI), and EverX Flow (XFLW) were included in this in vitro study. The compositions of the materials are presented in Table 1. Sample size estimation was performed using G*Power software (Version 3.1; Heinrich Heine University, Düsseldorf, Germany). Surface roughness (Ra) was selected as the primary outcome for the power analysis. Based on the study by Alnasser et al. [17], which evaluated changes in the surface roughness of esthetic restorative materials following an acidic challenge, an effect size of f = 0.66 was used. Utilizing the F-test family (one-way ANOVA model) with α = 0.05 and a statistical power of 95%, the minimum required sample size was determined to be 24 specimens per material group.

**Figure 1:**
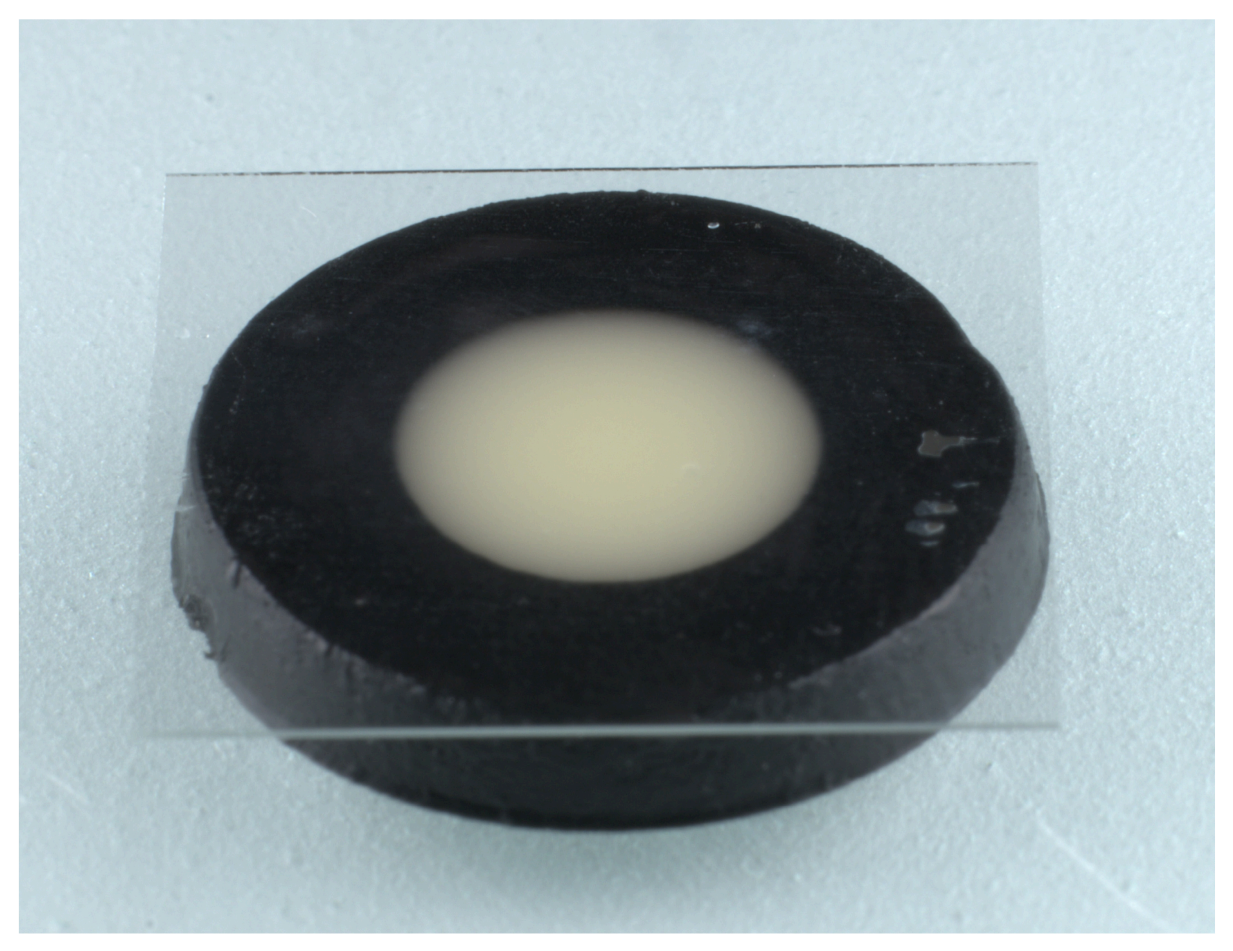
Representative shape and appearance of the disk-shaped specimens prepared from the tested flowable resin composites. All specimens were polymerized individually in standardized molds without contact between specimens. Samples were covered with glass plates on both sides to obtain flat surfaces and minimize oxygen inhibition.

A total of 96 disk-shaped specimens (*n* = 24 per material) were fabricated, with a diameter of 8 mm and a thickness of 2 mm. All materials were used in shade A2 (or universal shade, according to manufacturer instructions). The composite materials were placed into standardized silicone molds, covered with glass plates on both sides to obtain flat surfaces and minimize oxygen inhibition (Figure 1). Each specimen was individually light-cured for 20 s using an LED curing unit (Elipar DeepCure-S, 3M Deutschland GmbH, Seefeld, Germany; output intensity: 1200 mW/cm^2^). The light intensity was verified periodically using a radiometer, and the curing tip was positioned in direct contact with the glass surface. To standardize the specimen surfaces and simulate clinical finishing procedures, all specimens were polished by a single calibrated operator using Sof-Lex disks (3M Deutschland GmbH, Seefeld, Germany) from coarse to superfine grit under water cooling for 10 s per disk. A new disk was used after every five specimens to standardize abrasive efficiency. After polishing, all specimens were stored in distilled water at 37 °C for 24 h to allow post-polymerization and water equilibration before baseline measurements. After this storage period, the specimens were gently air-dried, and baseline surface roughness and color measurements were performed.

### 2.2. Surface Roughness

All measurements were performed by a blinded operator who was unaware of group allocation. Surface roughness (Ra, µm) was evaluated using a three-dimensional non-contact optical profilometer (Smartproof 5, Carl Zeiss Microscopy GmbH, Jena, Germany). Surface scans were obtained from the central region of each specimen under standardized magnification and measurement settings. Three measurements were recorded from different areas of each specimen, and the mean Ra value was used for statistical analysis.

### 2.3. Erosive Challenge and Coffee Aging

To simulate intrinsic erosive conditions, all specimens were immersed individually in 0.06 M hydrochloric acid (pH ≈ 1.2) for 48 h at 37 °C, following a previously validated protocol corresponding to approximately five years of cumulative gastric acid exposure [20]. For staining simulation, freshly prepared coffee solution (Nescafé Classic, Nestlé S.A., Vevey, Switzerland; 3.6 g/300 mL distilled water) was used. Specimens were immersed at 37 °C for 24 h, and the solution was renewed daily. Following acid and coffee exposure, specimens were then randomly allocated to subgroups using a computer-generated randomization list (*n* = 8 per subgroup):

- Toothbrushing (BR),
- Water flosser (WF),
- Mouthrinse (MR).

The details of each procedure are provided in Table 2.

### 2.4. Post-Treatment Measurements

**Figure 2:**
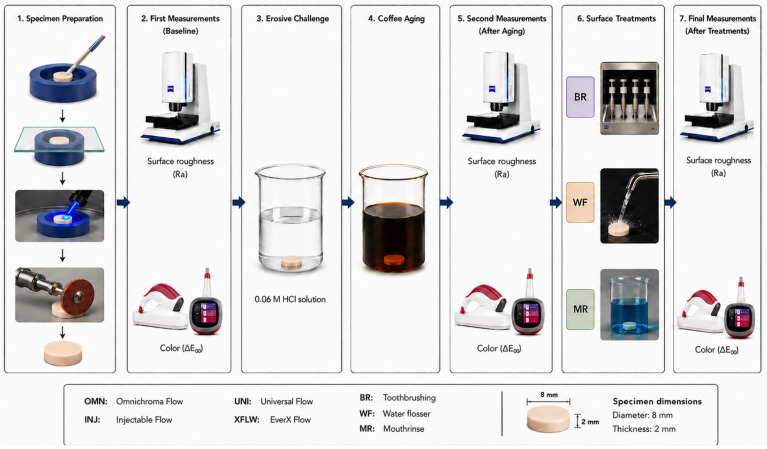
A schematic overview of the experimental design and workflow.

Surface roughness and color measurements were performed at three time points: baseline, after sequential gastric acid–coffee exposure, and after the assigned cleaning procedures. Color measurements were performed against a white background using a spectrophotometer (VITA Easyshade V, VITA Zahnfabrik H. Rauter GmbH & Co. KG, Bad Säckingen, Germany). Baseline color coordinates were recorded as L_0_*, a_0_*, and b_0_*. After sequential simulated gastric acid–coffee exposure, color coordinates were recorded as L_1_*, a_1_*, and b_1_*, and ΔE_0_ was calculated between baseline and post-exposure values. Therefore, ΔE_0_ represented the color change after the sequential erosive–staining challenge. Following the assigned cleaning procedures, final color coordinates were recorded as L_2_*, a_2_*, and b_2_*. ΔE_1_ was calculated between the post-exposure and post-cleaning values (L_1_*, a_1_*, b_1_* vs. L_2_*, a_2_*, b_2_*) to evaluate the color change associated with the cleaning procedures. Three consecutive measurements were obtained from each specimen, and the mean value was used to calculate ΔE_00_ according to the CIEDE2000 formula [23]. A schematic overview of the experimental design is presented in Figure 2. After each experimental stage, specimens were rinsed with distilled water to remove residual solution and gently air-dried before measurement. Post-exposure measurements were performed immediately after the sequential gastric acid–coffee exposure and after the assigned cleaning procedures under standardized conditions.

### 2.5. Scanning Electron Microscopy (SEM)

**Figure 3:**
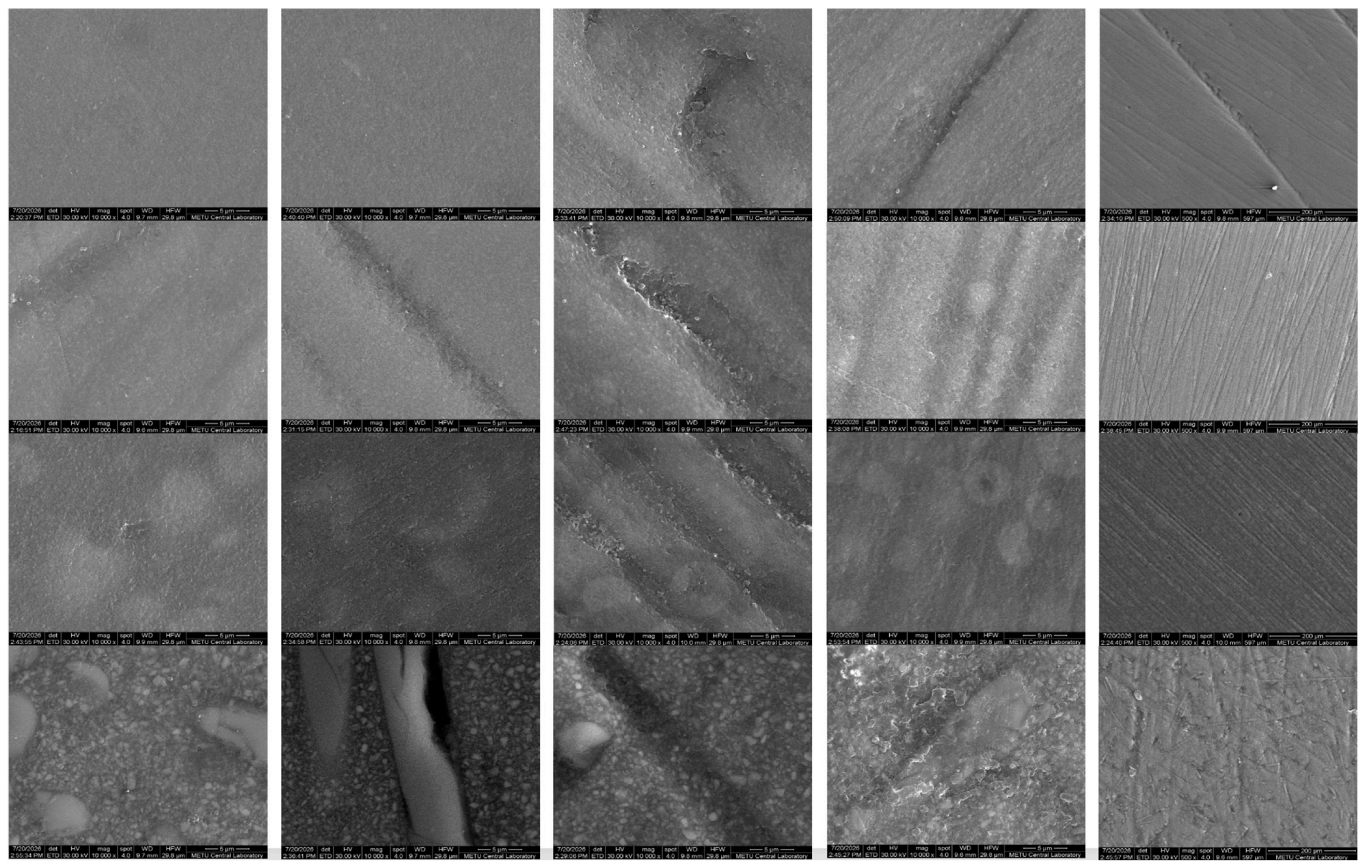
Representative SEM images showing the effects of the experimental procedures on the surface morphology of the composite materials. Each row corresponds to a composite group: (1) UNI, (2) INJ, (3) OMN, and (4) XFLW. The columns represent the different surface conditions and exposures: the first column shows the untreated composite surfaces; the second column shows the surfaces after gastric acid (GA) exposure; the third column shows the surface changes following toothbrushing; and the fourth column shows the surfaces after mouthrinse exposure. Images in the first four columns were obtained at (×10,000) magnification. The fifth column presents lower-magnification images (×500) demonstrating the broader surface abrasion pattern caused by toothbrushing.

For qualitative surface evaluation, five additional specimens from each experimental group were prepared to represent the different experimental stages. Before SEM examination, the specimens were sputter-coated with a 3 nm-thick gold–palladium layer and subsequently examined using a Quanta 400F field-emission scanning electron microscope (FE-SEM; FEI Company, Hillsboro, OR, USA) at ×500 and ×10,000 magnifications. The SEM images were used for descriptive purposes only and were not included in the statistical analysis. Figure 3 illustrates the surface morphological changes associated with the applied experimental procedures.

### 2.6. Statistical Analysis

Statistical analyses were performed using R software (version 4.3.1; R Foundation for Statistical Computing, Vienna, Austria). Data normality was assessed using the Shapiro–Wilk test and homogeneity of variance using Levene’s test. All data were normally distributed according to the Shapiro–Wilk test (*p* > 0.05). Surface roughness data were analyzed using a three-way mixed ANOVA. Material type and cleaning procedure were included as between-subject factors, while measurement stage was included as the within-subject repeated-measures factor with three levels: baseline (Ra0), after sequential gastric acid–coffee exposure (RaGC), and after the assigned cleaning procedure (Ra1). The assumptions of normality and homogeneity of variance were assessed using the Shapiro–Wilk and Levene tests, respectively. The sphericity assumption for the repeated-measures factor was evaluated using Mauchly’s test. When the sphericity assumption was violated, Greenhouse–Geisser correction was applied. Generalized eta-squared (ges) values were reported as effect sizes. Color change data were analyzed using two-way ANOVA. Bonferroni-adjusted post hoc comparisons were applied when appropriate. Statistical significance was set at α = 0.05.

## 3. Results

Following cleaning procedures, post-treatment Ra values ranged from 0.21 ± 0.02 µm (OMN-WF) to 0.92 ± 0.17 µm (INJ-BR). In most materials, BR subgroups showed higher Ra values than WF subgroups, while MR values were generally intermediate. Percentage increases ranged from 18.6% (OMN-WF) to 99.3% (XFLW-MR). Three-way mixed ANOVA revealed significant main effects of material, cleaning procedure, and measurement stage on surface roughness (*p* < 0.001). Significant interaction effects were also found for material × measurement stage, cleaning method × measurement stage, and material × cleaning method × measurement stage (Table 3).

Following cleaning procedures, Ra values further increased in most groups. Toothbrushing generally resulted in the highest surface roughness values, particularly in UNI and INJ. The highest post-treatment Ra value was observed in INJ-BR (0.92 ± 0.17 µm), while the lowest value was found in OMN-WF (0.21 ± 0.02 µm). Overall, OMN showed the lowest roughness values, whereas INJ exhibited the highest susceptibility to surface roughening.

**Figure 4:**
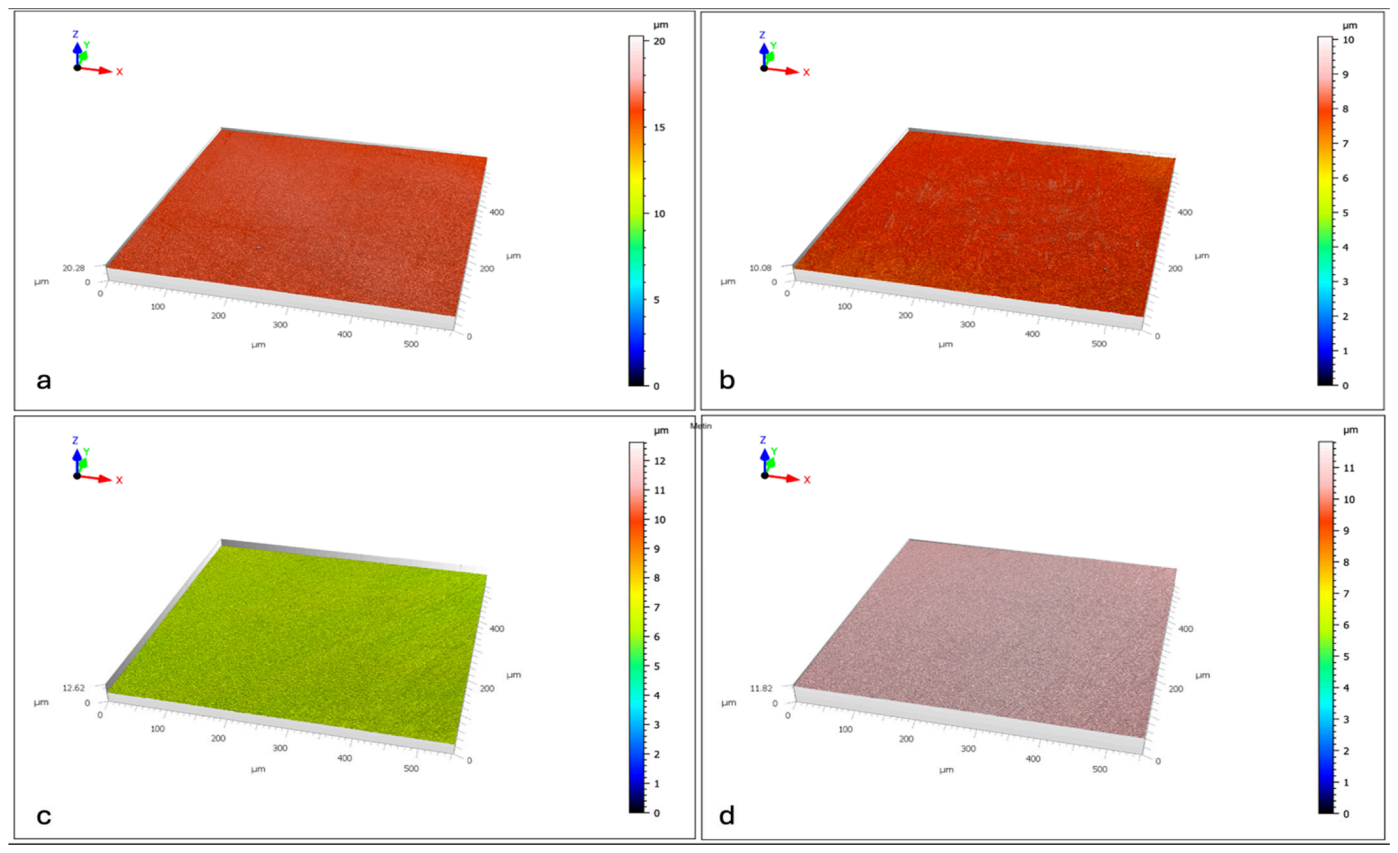
3D images of surface roughness of composite materials. (**a**) Universal flo group. (**b**) EverX Flow, (**c**) Omnichroma Flow, and (**d**) G-ænial Universal Injectable.

In terms of initial surface roughness (Ra_0_), the lowest value was observed in the OMN group (0.09 ± 0.03), whereas the highest value was recorded in the INJ group (0.17 ± 0.12). Following gastric acid and coffee exposure, Ra_GC_ values increased in all materials compared with Ra_0_. The highest Ra_GC_ value was detected in the INJ group (0.54 ± 0.12), while the lowest value was found in the OMN group (0.19 ± 0.05). When post-cleaning surface roughness (Ra_1_) was evaluated, the highest values in most materials were obtained in the BR group. The highest Ra_1_ value was identified in the INJ-BR group (0.92 ± 0.17), whereas the lowest value was observed in the OMN-WF group (0.21 ± 0.02). Ra_1_ values ranged from 0.46 ± 0.14 to 0.77 ± 0.10 in the UNI group, from 0.63 ± 0.10 to 0.92 ± 0.17 in the INJ group, from 0.21 ± 0.02 to 0.36 ± 0.14 in the OMN group, and from 0.39 ± 0.07 to 0.50 ± 0.07 in the XFLW group (Figure 4).

In terms of percentage change (%Δ), the greatest increase was observed in the XFLW-MR group (99.3%), whereas the smallest increase was found in the OMN-WF group (18.6%). Overall, the OMN group exhibited the lowest surface roughness values, while the INJ group demonstrated comparatively higher roughness values. The effects of material, cleaning procedure, and measurement stage on surface roughness were evaluated using three-way mixed ANOVA, and the results are presented in Table 4. Material showed the strongest effect among the tested factors. The color change values of the materials and treatment groups are presented in Table 5. In terms of color change after gastric acid and coffee exposure (ΔE_0_), the highest values were observed in the UNI group (3.99 ± 0.30–4.15 ± 0.36), whereas the lowest values were recorded in the OMN group (1.67 ± 0.36–1.78 ± 0.27). No significant differences in ΔE_0_ were found among the treatment applications within the UNI and INJ groups (*p* > 0.05). However, in the XFLW group, the WF application showed significantly higher values than the MR application (*p* < 0.05).

Color change values after cleaning (ΔE_1_) generally decreased compared with ΔE_0_ values. In the UNI group, the highest ΔE_1_ value was observed in the MR application (3.64 ± 0.45), while the lowest value was recorded in the BR application (1.42 ± 0.65). In the INJ group, the highest value was found in the MR subgroup (3.09 ± 0.60), whereas the lowest value was detected in the WF subgroup (0.87 ± 0.43). In the OMN group, ΔE_1_ values ranged from 0.70 ± 0.30 to 1.72 ± 0.49, while in the XFLW group, the highest value was obtained in the MR application (2.26 ± 0.45). Overall, the lowest post-cleaning color change values were observed in the OMN and WF groups.

According to the results of the two-way analysis of variance, the material factor had a statistically significant effect on the dependent variable (F = 151.18, *p* < 0.001) (Table 6). In contrast, the main effect of the treatment factor was not statistically significant (F = 1.26, *p* = 0.288). However, the material × treatment interaction was statistically significant (F = 2.52, *p* = 0.027). This finding indicates that the effect of the treatments varied according to the type of material.

## 4. Discussion

The present study evaluated the combined effects of sequential simulated gastric acid exposure followed by coffee immersion and various oral hygiene procedures on the surface and optical properties of flowable resin composites. Surface roughness significantly increased following gastric acid and coffee exposure across all materials, and the subsequent response to cleaning protocols was found to be material-dependent. While material type and its interaction with cleaning procedures significantly influenced color stability, the main effect of the cleaning procedure alone was not statistically significant. Therefore, the null hypothesis was partially rejected, specifically regarding the significant influence of material type and the interaction effects observed.

The increase in surface roughness after sequential gastric acid–coffee exposure is consistent with previous studies reporting that erosive conditions adversely affect resin-based restorative materials [24]. Under the sequential erosive–staining challenge, degradation of the resin matrix and the filler–matrix interface may lead to filler exposure, microcrack formation, and increased surface irregularities [25,26]. In the present study, all materials exhibited higher Ra values after gastric acid and coffee exposure compared to baseline (*p* < 0.001), indicating that acidic conditions may compromise surface integrity.

From a clinical perspective, surface roughness is also relevant for bacterial retention. A threshold value of approximately Ra = 0.2 µm has been associated with increased plaque accumulation [27]. In the present study, all materials exhibited baseline Ra values below this threshold. However, after gastric acid and coffee exposure, UNI, INJ, and XFLW exceeded this value, whereas OMN remained close to the threshold. These findings may suggest that OMN maintained surface smoothness more effectively under the sequential erosive–staining challenge, whereas INJ showed greater susceptibility to post-exposure roughening.

Following cleaning procedures, surface roughness increased further in most groups. Toothbrushing generally resulted in higher Ra values compared to other cleaning methods, particularly in UNI and INJ. This may be attributed to the abrasive effect of toothbrush bristles and toothpaste slurry on surfaces previously altered by the sequential gastric acid–coffee exposure. As emphasized in previous studies, erosion–abrasion interactions should be considered when evaluating surface degradation [28]. Therefore, toothbrushing performed after the sequential erosive–staining exposure may contribute to additional surface roughening.

Flowable resin composites were selected because of their increasing use in minimally invasive restorative procedures, cervical and buccal defects, small anterior and posterior restorations, and areas requiring enhanced adaptation and esthetic integration. These indications are clinically relevant in patients exposed to erosive conditions, such as gastroesophageal reflux disease, where superficial defects and restoration margins may be repeatedly challenged by acidic and staining agents. The tested materials were chosen to represent contemporary flowable composite technologies with distinct structural characteristics: Omnichroma Flow contains supra-nano spherical SiO_2_-ZrO_2_ fillers and is designed as a single-shade material; Universal Flo has a Bis-GMA-free nanohybrid formulation; G-ænial Universal Injectable is a highly filled injectable composite containing barium glass fillers with full-coverage silane coating; and EverX Flow is a short fiber-reinforced flowable composite. Therefore, the selected materials allowed comparison of different filler architectures, resin matrix formulations, and reinforcement strategies under the same sequential erosive–staining and cleaning protocol [2,12].

The material-dependent differences in surface roughness may be associated with variations in filler loading, filler size, filler morphology, resin matrix composition, and filler–matrix coupling. Filler characteristics are known to influence the mechanical and surface behavior of dental composites [29]. In the present study, OMN showed the lowest roughness values both initially and after the experimental procedures. This may be related to its high filler content and uniformly distributed supra-nano spherical SiO_2_-ZrO_2_ fillers, as shown in Table 1. In single-shade resin composites, uniformly sized supra-nano spherical fillers have been associated with structural color behavior and favorable optical adaptation [30].

Although this reference primarily relates to optical behavior, the relatively homogeneous filler structure of OMN may also have contributed to the lower roughness values observed in the present study. In contrast, INJ showed the highest roughness after gastric acid and coffee exposure and toothbrushing. Although this material contains barium glass fillers with full-coverage silane coating, its greater roughness may be related to differences in resin matrix composition, filler distribution, and the susceptibility of the filler–matrix interface to acidic and abrasive challenges. The filler–resin interface is considered a critical region for stress transfer and crack propagation in dental composites [31]. Under sequential erosive–staining and hydrolytic conditions, degradation of this interface may lead to filler debonding, surface defects, and increased roughness [25]. In addition, resin matrix composition may influence water sorption, hydrolytic stability, and matrix softening. Therefore, the higher roughness observed in INJ may reflect the combined effects of exposure-related matrix degradation, filler–matrix interface weakening, and subsequent abrasive wear.

The behavior of XFLW may be related to its short fiber-reinforced structure. Although fiber incorporation can improve mechanical properties, the heterogeneous distribution of fibers and fillers may influence surface degradation under combined erosive and abrasive conditions. In the present study, XFLW showed considerable increases in surface roughness after cleaning procedures, which may be associated with differential wear between the resin matrix and reinforcing components [32,33].

Color stability findings also demonstrated clear material-dependent differences. Color changes were interpreted according to CIEDE2000 perceptibility and acceptability thresholds. A ΔE_00_ value of 0.8 has been reported as the 50:50% perceptibility threshold, whereas 1.8 is considered the 50:50% acceptability threshold in dentistry [34]. In the present study, the color changes observed after gastric acid and coffee exposure exceeded the perceptibility threshold in all materials and exceeded the acceptability threshold in most groups. UNI showed the highest ΔE_00_ values after gastric acid and coffee exposure, ranging from 3.99 ± 0.30 to 4.15 ± 0.36. INJ and XFLW also exceeded the acceptability threshold, with ΔE_00_ values ranging from 3.11 ± 0.41 to 3.40 ± 0.43 and from 2.59 ± 0.32 to 3.28 ± 0.51, respectively. In contrast, OMN showed ΔE_00_ values between 1.67 ± 0.36 and 1.78 ± 0.27, remaining close to the acceptability threshold but generally below it.

The clinically unacceptable discoloration observed in INJ may be explained by its increased surface roughness and material-dependent susceptibility to the combined erosive–staining challenge rather than by a single compositional factor. As discussed above, INJ showed the highest Ra values after gastric acid and coffee exposure and toothbrushing, indicating that its surface was more affected by the combined erosive and abrasive challenge. The resulting surface irregularities may have provided micro-retentive areas for coffee chromogens, thereby increasing pigment adsorption. Surface condition and storage media have been shown to influence the color stability of resin-based composites [19]. In addition, degradation of the resin matrix and filler–matrix interface under acidic and hydrolytic conditions may contribute to surface defects and roughening [25]. This mechanism may be particularly relevant for INJ, which contains a methacrylate-based resin matrix and barium glass fillers with full-coverage silane coating, as shown in Table 1.

The lower color change observed in OMN may be associated with its lower surface roughness and more homogeneous supra-nano spherical filler structure. Reduced surface irregularities may limit pigment retention on the material surface. In addition, its filler structure may contribute to favorable optical behavior in single-shade composites [35]. Together, these factors may help explain the lower ΔE_00_ values observed. However, color stability is multifactorial and may also be influenced by resin matrix composition, filler morphology, water sorption, and filler–matrix interface stability [25,35].

After cleaning procedures, ΔE_00_ values generally decreased, suggesting partial removal of extrinsic staining. However, this reduction was both material- and procedure-dependent. While some groups approached or fell below the CIEDE2000 acceptability threshold, the mouthrinse groups of UNI, INJ, and XFLW remained above this limit [34]. These findings indicate that although cleaning procedures may reduce visible discoloration, their effectiveness varies depending on the material and may not fully restore clinically acceptable color stability.

The relatively higher residual discoloration observed after mouthrinse application may be related to the absence of a direct mechanical cleaning effect. Unlike toothbrushing or water flossing, mouthrinse immersion may have limited ability to remove pigments retained within surface irregularities. In contrast, water flossing resulted in lower color change values in several groups, possibly by removing superficial pigments without abrasive contact. A previous study has also shown that water-jet flossing may influence surface roughness and color stability of resin-based composites [22]. However, these effects may vary depending on application parameters such as pressure, duration, and nozzle distance.

From a clinical perspective, these findings suggest that the performance of flowable resin composites under erosive and cleaning challenges is material dependent. In patients exposed to recurrent acidic conditions, clinicians should consider both material selection and oral hygiene practices [5]. Since most materials exceeded the Ra = 0.2 µm threshold after experimental procedures, individualized hygiene recommendations are important [27]. In particular, aggressive toothbrushing immediately after acidic exposure may increase surface roughness, whereas less abrasive cleaning approaches may help preserve surface integrity [3,22].

This study has several limitations. The in vitro design cannot fully replicate the complex oral environment, including saliva, biofilm, thermal changes, and mechanical loading. In addition, the continuous gastric acid and coffee exposure protocol may not accurately reflect intermittent clinical reflux conditions. However, the equivalence of in vitro simulation protocols to specific clinical time periods should be interpreted cautiously, as oral conditions are highly dynamic. Although SEM analysis was performed to support the surface roughness findings, AFM and microhardness testing were not included. Future studies should include more clinically relevant aging models and additional surface characterization methods to further clarify the degradation mechanisms. Within these limitations, gastric acid and coffee exposure increased surface roughness and affected color stability in a material-dependent manner. OMN demonstrated more favorable surface and optical properties, whereas INJ showed greater susceptibility to roughness increase. UNI exhibited higher discoloration values after chemical exposure. These findings highlight the importance of material selection and appropriate cleaning strategies in patients exposed to erosive conditions.

## 5. Conclusions

Sequential simulated gastric acid–coffee exposure increased surface roughness and caused material-dependent color changes in the tested flowable resin composites. Omnichroma Flow generally showed more favorable surface and optical stability, whereas G-ænial Universal Injectable exhibited greater susceptibility to surface roughening and Universal Flo showed higher discoloration after exposure. From a clinical standpoint, delaying toothbrushing after erosive–staining exposure and selecting materials with higher resistance to surface degradation may improve long-term restorative outcomes.

## Figures and Tables

**Table 1:** Chemical composition, filler characteristics, and manufacturers of the flowable resin composites evaluated.

Material	Resin Matrix	Filler/Formulation Characteristics	Filler Content (wt%)	Manufacturer/Country
Omnichroma Flow	UDMA/TEGDMA with photoinitiator	260 nm SiO_2_-ZrO_2_ supra-nano spherical fillers (sol–gel)	~79 wt% (68 vol%)	Tokuyama Dental Corporation, Tpkyo, Japan (LOT: 038E15)
Universal Flo	Bis-GMA-free resin matrix	~200 nm strontium glass fillers (nanohybrid structure)	~69 wt% (50 vol%)	GC Corporation, Tokyo Japan (LOT: 2502181)
G-ænial Universal Injectable	Methacrylate-based monomers (unspecified)	150 nm barium glass fillers with Full-coverage Silane Coating (FSC)	~69 wt%	GC Corporation, Tokyo Japan (LOT: 2403221)
EverX Flow	Bis-MEPP, TEGDMA, UDMA	~25 wt% short E-glass fibers (6 µm × 140 µm) + 42–52 wt% barium glass fillers + silica (trace)	~70 wt%	GC Corporation, Tokyo Japan (LOT: 2311131)

**Table 2:** Parameters and clinical equivalencies of the simulated oral hygiene procedures.

Tooth Brushing (BR)	Toothbrushing simulation was performed using an automatic brushing machine under a constant vertical load of 200 g at 120 strokes/min. A slurry of toothpaste and distilled water (1:2, *w*/*w*) was used. Each specimen underwent 50,000 strokes, corresponding to approximately five years of brushing [21].
Water Flosser (WF)	Oral irrigator simulation was performed using a countertop water flosser (Philips Sonicare Power Flosser 3000, Philips Consumer Lifestyle B.V., Drachten, The Netherlands) Specimens were fixed in a custom holder, and the nozzle was positioned perpendicular to the surface (≈90°) at a distance of 2 mm. A continuous water jet at medium pressure was applied for 30 min per specimen. The procedure was repeated once for each sample, after which specimens were rinsed with distilled water and gently air-dried prior to subsequent analyses. The 30 min application period was selected to approximate long-term simulated water-flosser use over a five-year period [22].
Mouthrinse (MR)	Specimens were immersed in Listerine mouthrinse (Johnson & Johnson Consumer Inc., Skillman, NJ, USA) at 37 °C for 120 h, corresponding to approximately five years of daily clinical use (2 min/day). The solution was renewed every 24 h. At the end of the immersion period, all specimens were rinsed with distilled water and gently air-dried [18].

**Table 3:** Surface roughness values of the tested flowable resin composites at baseline, after sequential gastric acid–coffee exposure, and after cleaning procedures.

Materials	Ra_0_ (Initial)	RaGC	Cleaning Method (CM)	Ra_1_	%Δ (Effect of Roughness)
UNI	0.15 ± 0.12 ^A^	0.46 ± 0.15 ^B^	BR	0.77 ± 0.10 ^Ca^	58.2%
			WF	0.46 ± 0.14 ^Bb^	22.2%
			MR	0.65 ± 0.12 ^Ca^	51.0%
INJ	0.17 ± 0.12 ^A^	0.54 ± 0.12 ^B^	BR	0.92 ± 0.17 ^Ca^	59.2%
			WF	0.63 ± 0.10 ^Bb^	23.1%
			MR	0.76 ± 0.15 ^Ca^	48.6%
OMN	0.09 ± 0.03 ^A^	0.19 ± 0.05 ^B^	BR	0.36 ± 0.14 ^Ba^	79.3%
			WF	0.21 ± 0.02 ^Ab^	18.6%
			MR	0.29 ± 0.10 ^Ba^	58.0%
XFLW	0.14 ± 0.05 ^A^	0.24 ± 0.06 ^B^	BR	0.50 ± 0.07 ^Ca^	91.6%
			WF	0.39 ± 0.07 ^Cb^	80.6%
			MR	0.44 ± 0.07 ^Ca^	99.3%

Ra_0_: baseline surface roughness; RaGC: surface roughness after sequential gastric acid–coffee exposure; Ra_1_: surface roughness after the assigned cleaning procedure; BR: toothbrushing; MR: mouthrinse; WF: water flosser; Data are presented as mean ± standard deviation. Different uppercase letters indicate statistically significant differences among measurement stages.

**Table 4:** Three-way mixed ANOVA results evaluating the effects of material type, cleaning procedure, and measurement stage on surface roughness.

	dfn	dfd	F	*p*	ges (Effect Size)
Materials	3.00	84.0	72.551	<0.001	0.609
cleaning procedure	2.00	84.0	11.513	<0.001	0.141
Measurement stage	1.81	152.3	615.558	<0.001	0.745
Material × cleaning procedure	6.00	84.0	0.647	0.692	0.27
Material × measurement stage	5.44	152.3	37.501	<0.001	0.348
Cleaning procedure × measurement stage	3.63	152.3	18.957	<0.001	0.153
Material × cleaning procedure × measurement stage	10.88	152.3	1.876	0.047	0.051

dfn: numerator degrees of freedom; dfd: denominator degrees of freedom; ges: generalized eta-squared effect size. The Greenhouse–Geisser correction was applied when the sphericity assumption was violated.

**Table 5:** Color change values (ΔE_00_) of tested materials after sequential gastric acid–coffee exposure and after cleaning procedures.

Materials	App	ΔE_0_	ΔE_1_
UNI	BR	4.15 ± 0.36 ^aA^	1.42 ± 0.65 ^bAB^
	MR	3.99 ± 0.30 ^aA^	3.64 ± 0.45 ^aA^
	WF	4.08 ± 0.53 ^aB^	2.25 ± 0.73 ^bB^
INJ	BR	3.24 ± 0.29 ^aB^	1.66 ± 0.64 ^abA^
	MR	3.40 ± 0.43 ^aB^	3.09 ± 0.60 ^aA^
	WF	3.11 ± 0.41 ^aB^	0.87 ± 0.43 ^bC^
OMN	BR	1.72 ± 0.37 ^aC^	0.95 ± 0.37 ^abB^
	MR	1.67 ± 0.36 ^aC^	1.72 ± 0.49 ^aB^
	WF	1.78 ± 0.27 ^aC^	0.70 ± 0.30 ^bC^
XFLW	BR	2.70 ± 0.44 ^abB^	1.25 ± 0.66 ^bAB^
	MR	2.59 ± 0.32 ^bB^	2.26 ± 0.45 ^aB^
	WF	3.28 ± 0.51 ^aB^	1.06 ± 0.48 ^bC^

Within the same row, different lowercase letters indicate statistically significant differences between ΔE_0_ and ΔE_1_. Within the same column, different uppercase letters indicate statistically significant differences among materials for the same cleaning procedure. (*p* < 0.05). ΔE_0_: color change between baseline and after sequential gastric acid–coffee exposure; ΔE_1_: color change between after sequential gastric acid–coffee exposure and after cleaning procedures; BR: toothbrushing; MR: mouthrinse; WF: water flosser.

**Table 6:** Results of two-way ANOVA for post-cleaning color change (ΔE_1_).

	df	SS	MS	F	*p*
Materials	3	68.840	22.947	151.18	<0.001
cleaning procedure	2	0.383	0.192	1.26	0.288
Material × cleaning procedure	6	2.292	0.382	2.52	0.027

df: degrees of freedom; SS: sum of squares; MS: mean square. Statistical significance was set at α = 0.05.

## Data Availability

The data supporting the findings of this study are available from the corresponding author upon reasonable request.

## Abbreviations

The following abbreviations are used in this manuscript:

GERD	Gastroesophageal reflux disease
OMN	Omnichroma Flow
INJ	Injectable Flow
UNI	Universal Flo
XFLW	EverX Flow
BR	Tooth Brushing
MR	Mouthrinse
WF	Water Flosser
